# 窄带成像联合自荧光支气管镜在肺癌诊断中的价值

**DOI:** 10.3779/j.issn.1009-3419.2013.06.05

**Published:** 2013-06-20

**Authors:** 众博 陈, 亦鸣 虞, 士芳 孙, 红映 马, 巧丽 张, 丹 吕, 碧炯 王, 超 曹, 群力 丁, 在春 邓

**Affiliations:** 315020 宁波，宁波大学医学院附属医院呼吸内科 Department of Respiratory Diseases, Affiliated Hospital of Ningbo University Medical College, Ningbo 315020, China

**Keywords:** 窄带成像, 自荧光成像, 肺肿瘤, 诊断, Narrow-band imaging (NBI), Autofluorescence imaging (AFI), Lung neoplasms, Diagnosis

## Abstract

**背景与目的:**

窄带成像（narrow-band imaging, NBI）与自荧光成像（autofluorescence imaging, AFI）是近几年临床诊断肺癌的支气管镜新技术。本研究旨在研究这两种技术的结合是否能提高肺癌诊断的敏感性和特异性。

**方法:**

该项目共纳入137例疑似肺癌患者，所有患者的检查均基于Olympus Evis Lucera电子支气管镜系统，依次进行白光支气管镜（white light bronchoscopy, WLB）、窄带成像、自荧光成像检查，在每位患者镜下异常部位至少取3块组织送检。

**结果:**

WLB的敏感性、特异性分别为56.6%和62.5%；NBI成像的敏感性、特异性分别为71.3%和75.0%；AFI敏感性、特异性分别为82.2%和25.0%；NBI联合AFI的敏感性、特异性分别为94.6%和87.5%。NBI+AFI与AFI相比，两者敏感性及特异性均有统计学差异（*P* < 0.01），与单用NBI相比，两者敏感性及特异性亦有统计学差异（*P* < 0.05）。

**结论:**

NBI或AFI比WLB在肺癌诊断方面具有更好的敏感性，且联合使用NBI+AFI比其它任何一种单用技术具有更高的敏感性和特异性优势。

## Abstract (en)

**Background and objective:**

Narrow-band imaging (NBI) and autofluorescence imaging (AFI) are new bronchoscopy technologies that are important in lung cancer diagnosis. The aim of this study is to determine whether or not the combination of these two technologies can improve sensitivity and specificity of lung cancer diagnosis.

**Methods:**

A total of 137 patients who manifested symptoms of lung cancer were investigated in this project. All of the examinations were performed based on an Olympus Evis Lucera bronchoscopy system. The patients were examined by white light bronchoscopy (WLB), NBI and AFI. At least three biopsies from body parts visualized as lesions were obtained from each patient.

**Results:**

WLB sensitivity and specificity were 56.6% and 62.5%, respectively. NBI sensitivity and specificity were 71.3% and 75.0%, respectively. AFI sensitivity and specificity were 82.2% and 25.0%, respectively. The sensitivity and the specificity of the combined NBI and AFI were 94.6% and 87.5%, respectively. The sensitivity and the specificity of the combined NBI and AFI were significantly higher than those of AFI alone (*P* < 0.01). Likewise, the sensitivity and the specificity of the combined NBI and AFI were significantly higher than those of NBI alone (*P* < 0.05).

**Conclusion:**

NBI or AFI exhibited higher sensitivity of lung cancer diagnosis than WLB. Combined NBI and AFI also showed significantly higher sensitivity and specificity than NBI or AFI.

窄带成像（narrow-band imaging, NBI）和自荧光成像（autofluorescence imaging, AFI）是支气管镜中相对较新的技术，尤其NBI是一种新兴的特殊光支气管镜技术。NBI是利用滤光器过滤内镜光源所发出的红蓝绿光波中的宽带光谱，仅留下窄带光谱，其蓝色光谱波长为390 nm-445 nm，而绿色光谱波长为530 nm-550 nm，其优势不仅能够精确观察支气管粘膜上皮形态，还可观察上皮血管网的形态^[1, 2]^。这两种技术目前已被广泛用于支气管镜下对癌前病变和早期中央型肺癌（early central lung cancer, ECLC）的诊断^[3, 4]^。大多数临床试验的研究结果表明AFI及NBI的使用对于诊断肺癌具有潜在益处，尤其是检测癌前病变。除了检测早期肺癌，AFI及NBI还是肺癌术前评估及术后复查的重要检查工具^[7-9]^。这些技术的主要优点是能使镜下异常粘膜清晰且差异化表现。虽然从普通白光显像（white light imaging, WLI）切换到另一种模式非常方便，但AFI与NBI使用的却是不同的支气管镜，需要在同一患者身上使用两种不同的支气管镜，增加了检查时间且需要至少两次进出声门的过程^[10]^。这可能是目前AFI及NBI联合诊断肺癌文献较少的主要原因。本研究旨在评估NBI+AFI联合使用在诊断肺癌中的价值。

## 1 资料与方法

### 1.1 研究病例

此项研究为前瞻性、非随机的临床研究，对宁波大学医学院附属医院2011年7月-2012年12月门诊或住院期间高度怀疑肺部肿瘤的患者进行支气管镜检查。研究获医院伦理委员会的批准。纳入标准：年龄 > 18岁，胸部CT扫描高度怀疑为肺癌的患者。排除标准：无法耐受支气管镜检查、近期心肌梗死、未解决的凝血功能障碍、不稳定型心绞痛、慢性心力衰竭、近3个月内脑梗塞或脑出血、未控制的心律不齐或高血压的患者。

### 1.2 设计与检查方法

所有患者必须有胸部CT扫描、肺功能检查、血气分析、血常规、血生化、凝血功能、HIV、RPR、HbsAg结果。161例拟行支气管镜检查的患者最终有137例符合纳入标准，所有符合条件的患者均同意该检查，了解并签署知情同意书。

术前准备：使用2%利多卡因气道麻醉，同时心电监护监测动脉血压、氧饱和度、心率。在研究中使用的设备包括BF-F260（AFI）、BF-1T260（NBI）和视频处理器单元EVIS LUCERA光谱（CV-260SL）（Olympus公司，日本东京）。AFI和NBI显像于19英寸的液晶显示器（OEV-191）。

依次使用白光支气管镜（white light bronchoscopy, WLB）、NBI和AFI观察气管、各级支气管。AFI显像支气管粘膜为棕红色或品红色的区域定义为阳性，而阴性的区域为绿色。根据NBI显像异常的描述^[11, 12]^，支气管粘膜表现为斑点、粘膜迂回曲折或血管走形突然中断为阳性。对3种方法中任一模式所发现的粘膜异常部位拟行组织学活检，并标明发现异常的模式、部位。不同部位使用单独的活检钳，不同部位标本分在不同的活检瓶中，至少取3块及以上合格大小的组织（目测单个标本大小≥2 mm）。通常等3种模式观察完毕，同时记录哪种模式下在哪些部位有异常然后再取活检。由于理论上AFI敏感性最高，因此AFI为最后使用的检查模式，亦在该模式下取病理活检，避免再次更换支气管镜。如出现多个部位粘膜异常，为避免活检出血导致镜下视野不清，活检顺序按“由远及近”的原则进行。所有镜下活检标本送到至少有10年以上病理诊断经验的病理医生处进行镜下诊断且并不告知临床诊断结果。

### 1.3 统计学方法

所有的统计分析均采用SPSS for Mac版本20.0（SPSS公司）。采用Mean±SD表述计量资料，卡方检验比较诊断的敏感性和特异性。*P* < 0.05为差异有统计学意义。

## 2 结果

### 2.1 一般情况

137例行支气管镜检查的患者中，有105例（76.6%）男性和32例（23.4%）女性。患者的平均年龄61岁±10岁。吸烟者94例（68.6%），其中有29例（30.9%）已戒烟，从不吸烟者43例。

### 2.2 病理诊断及分型

137例患者中最终病理证实恶性肿瘤的129例。最常见的肿瘤类型为肺鳞状细胞癌81例（62.8%），腺癌33例（25.6%），小细胞肺癌13例（10.1%），未分型癌2例（1.6%），所有病理分型结果均以支气管镜下活检报告为评估标准，137例中24例同时有手术病理且与支气管镜下活检病理一致。最常见的肿瘤部位为右下叶背段23例（17.8%），其次为左下叶基底段17例（13.1%）及右上叶17例（13.2%），详见表 1。

**1:** 肿瘤部位 Tumor localizations

Localization	Frequency	Percent (%)
Right main bronchus	13	10.1
Right upper lobe bronchus	17	13.2
Intermediary bronchus	12	9.3
Right B6	23	17.8
Right basal bronchi (B7-B10)	15	11.6
Left main bronchus	9	7.0
Left upper lobe bronchus	6	4.7
Left B4-B5	5	3.9
Left B6	12	9.3
Left basal bronchi (B7-B10)	17	13.1
Total	129	100.0

### 2.3 不同方法敏感性、特异性比较

WLB、NBI、AFI三种技术中，AFI敏感性最高，而NBI则是三者中特异性最高的技术，联合NBI+AFI两种技术后，敏感性及特异性得到了明显提升（表 2）。NBI与WLB相比，相对敏感度和特异度分别为1.3和1.2；NBI与AFI相比，相对敏感度和特异度分别为0.9和3.0。NBI+AFI与单用AFI相比，相对敏感度和特异度分别为1.2和3.5；NBI+AFI与NBI相比，相对敏感度和特异度分别为1.03和1.05。NBI+AFI与AFI相比，两组敏感性（*P*=0.007）和特异性（*P* < 0.01）差异均有统计学意义；与单用NBI相比，两者敏感性（*P* < 0.01）和特异性（*P*=0.032）差异均有统计学意义。

**2:** WLB、NBI、AFI及NBI+AFI的敏感性、特异性比较 Sensitivity and specificity of WLB, NBI, AFI, and combination of NBI+AFI

Group	Sensitivity (%)	Specificity (%)
WLB	56.6	62.5
NBI	71.3	75.0
AFI	82.2	25.0
NBI+AFI	94.6	87.5
WLB: white light videobronchoscopy; NBI: narrow band imaging videobronchoscopy; AFI: autofluorescence imaging videobronchoscopy.		

## 3 讨论

如何早期诊断肺癌是提高肺癌患者生存率的重要因素。虽然近几年AFI广泛应用于临床并提高了对中央型肺癌诊断的敏感性，但因其特异性不高，导致在镜下观察时往往发现有多处支气管粘膜“异常”，难以甄别出真正需要取病理组织的部位。而NBI技术的出现则可以辅助AFI去发现那些异常部位是否存在“真正的粘膜异常”。NBI所能观察到的粘膜表面血管异常是AFI或WLB所不具备的。

本项研究的结果显示，NBI或AFI比WLB在肺癌诊断方面具有更好的敏感性，且联合使用NBI+AFI比其它任何一种单用技术具有更高的敏感性和特异性优势。通过NBI+AFI这两种技术的结合使用，AFI的特异性得到明显改善。在研究中发现，AFI镜下显示粘膜异常较为常见，往往在多个点位的支气管粘膜显示品红色，经活检发现特异性并不高。Yasufuku等^[3]^研究了AFI在ECLC中的诊断价值后发现，AFI不但在侵袭性癌（invasive carcinoma, IC）、原位癌（carcinoma *in situ*, CIS）有显影异常，且在所有程度的粘膜异型增生均有色泽改变。而我们在研究中亦发现，如仔细观察部分老年患者或支气管炎症患者的支气管壁，可出现支气管纵型皱襞增粗，而增粗的纹理间可见淡品红色异常显影。由此认为AFI在所有支气管粘膜异常增厚的情况下均会出现显影异常。

如在AFI发现异常的基础上再使用NBI观察AFI所发现的异常粘膜，则会有令人兴奋的发现，真正具有粘膜癌变的部位可在NBI模式下表现为“芝麻点”样改变（图 1），这种特异性的表现在其它支气管镜模式下是无法被发现的。另外，NBI的技术特点还被用于检测微细异常支气管粘膜，NBI与AFI两者结合不但能检测ECLC，且在所有级别的炎症的诊断方面亦具有一定优势^[13]^。

**1:**
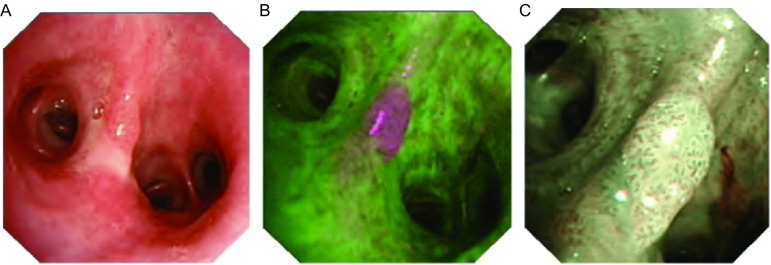
三种不同光谱下癌变支气管粘膜的显影。A：WLB显示左肺下叶间嵴水肿、肥厚；B：AFI显示间嵴品红色改变；C：NBI显示间嵴粘膜肥厚、隆起，表面血管异常，表现为“芝麻点”样改变。 Image of WLB, AFI and NBI. A: Irregularmucosa are identified at the bifurcation of the left lower lobe bronchi by WLB; B: The AFI image clearly is magenta at the abnormal area; C: In the NBI mode, atypical dotted vessels are visible. The biopsy showed squamous cell cancer.

对照Vincent等^[8]^的研究文献，在评估癌前病变方面，NBI与WLB的相对敏感度为1.63，而NBI的特异性为81%，高于WLB的64%，两者差异有统计学意义。而Herth等^[5]^的研究表明，NBI+AFI虽然比单用AFI或单用NBI提高了敏感性，但与NBI相比并没有明显的特异性优势，这点与我们的研究结果有所不同。进一步研究和评估NBI+AFI在诊断ECLC的敏感性和特异性是必要的，因目前相关文献仍较少，继续重复这方面的研究以便在临床实践中找出一定的应用特点。

本研究的主要缺陷是所有参与试验的支气管镜检查医师都具有丰富的支气管镜检查经验，所有参与检查的医师已经知晓两种检查方式的优势所在，会更加注意两者的镜下特点，而存在忽略WLB镜下粘膜细微变化的可能。然而在具体实践过程中，想要完全避免这种主观偏向是不可能的。其次，本研究的病例中大多数患者气道中可见明显的粘膜异常或实质性新生物，即没有癌前病变（中重度粘膜异型增生）的病例被发现，缺乏对癌前病变区域使用NBI观察的经验。再次，结合这两种检查技术在临床使用中较费时间，每次都需要重新检查一次气管、支气管，而且AFI与NBI并不在一条支气管镜上，检查过程中需要更换支气管镜。克服这个问题的理想解决方案就是期望将NBI、AFI这两种技术集合于一条支气管镜。

NBI和AFI技术在诊断肺癌方面比WLB具有更高的敏感性，NBI+AFI技术的组合可能优于WLB+AFI。未来的研究应着眼于NBI+AFI这两种技术在肺癌癌前病变及肿瘤浸润范围的评估工作。
